# Candidate effector proteins of the necrotrophic apple canker pathogen *Valsa mali* can suppress BAX-induced PCD

**DOI:** 10.3389/fpls.2015.00579

**Published:** 2015-07-27

**Authors:** Zhengpeng Li, Zhiyuan Yin, Yanyun Fan, Ming Xu, Zhensheng Kang, Lili Huang

**Affiliations:** State Key Laboratory of Crop Stress Biology for Arid Areas and College of Plant Protection, Northwest A&F UniversityYangling, China

**Keywords:** apple *Valsa* canker, secreted protein, cell death, virulence factor, plant–fungus interaction

## Abstract

Canker caused by the Ascomycete *Valsa mali* is the most destructive disease of apple in Eastern Asia, resulting in yield losses of up to 100%. This necrotrophic fungus induces severe necrosis on apple, eventually leading to the death of the whole tree. Identification of necrosis inducing factors may help to unravel the molecular bases for colonization of apple trees by *V. mali.* As a first step toward this goal, we identified and characterized the *V. mali* repertoire of candidate effector proteins (CEPs). In total, 193 secreted proteins with no known function were predicted from genomic data, of which 101 were *V. mali*-specific. Compared to non-CEPs predicted for the *V. mali* secretome, CEPs have shorter sequence length and a higher content of cysteine residues. Based on transient over-expression in *Nicotiana benthamiana* performed for 70 randomly selected CEPs, seven *V. mali* Effector Proteins (VmEPs) were shown to significantly suppress BAX-induced PCD. Furthermore, targeted deletion of *VmEP1* resulted in a significant reduction of virulence. These results suggest that *V. mali* expresses secreted proteins that can suppress PCD usually associated with effector-triggered immunity (ETI). ETI in turn may play an important role in the *V. mali*–apple interaction. The ability of *V. mali* to suppress plant ETI sheds a new light onto the interaction of a necrotrophic fungus with its host plant.

## Introduction

The Apple *Valsa* canker fungus *Valsa mali* is a necrotrophic pathogen inducing severe necrosis on apple. It is the most devastating pathogen of apple in Eastern Asia, causing severe yield losses each year (Lee et al., 2006; Li et al., 2013). This pathogen preferentially infects apple although it is also aggressive to other Rosaceae woody plants such as pear, crabapple, apricot and peach (Wang et al., 2011b; Zhou et al., 2014). *V. mali* is considered a necrotroph that completes its life cycle on dead plant cells killed prior to or during colonization (Ke et al., 2013; Yin et al., 2015). Particularly, genes involved in plant cell wall degradation and toxin synthesis are remarkably expanded in the *V. mali* genome and are commonly up-regulated during infection (Ke et al., 2014; Yin et al., 2015). However, it is becoming more and more evident that interactions between necrotrophs and their hosts are considerably more complex and subtle than previously thought. Functional analysis of the *V. mali* genome showed that 193 secreted proteins have no known annotation, of which about 101 are *V. mali*-specific. Given that phytopathogens often secrete a series of proteins into the host–pathogen interface to manipulate host cell physiology and ultimately promote infection, functional verification of these secreted proteins is of particular interest for identifying potential virulence factors.

Effectors play an important role in the host–pathogen interface during infection (Giraldo and Valent, 2013; Vleeshouwers and Oliver, 2014). It is generally accepted that biotrophs actively suppress programmed cell death (PCD) of the host, whereas necrotrophs are believed to promote cell death to enhance colonization (Donofrio and Raman, 2012; Mengiste, 2012). Typically, most of the known effectors secreted by necrotrophic fungi (e.g., *Parastagonospora nodorum*, *Pyrenophora tritici-repentis*, *Alternaria alternata,* or *Cochliobolus heterostrophus*) lead to effector-triggered susceptibility (ETS) resulting in host cell death (reviewed in Wang et al., 2014). Intriguingly, the effector protein SSITL of *Sclerotinia sclerotiorum* can suppress the jasmonic/ethylene (JA/ET) signaling pathway mediated resistance at an early stage of infection (Zhu et al., 2013). In addition, oxalic acid secreted by *S*. *sclerotiorum* inhibits host autophagy which constitutes an effective defense response in this necrotrophic fungus–plant interaction (Kabbage et al., 2013). These evidences suggest that effectors of necrotrophic fungi may also suppress host defense responses, rather than induce cell death.

Programmed cell death triggered in plants by the pro-apoptotic mouse protein BAX physiologically resembles PCD associated with defense-related HR (Lacomme and Santa Cruz, 1999). As a result, the ability to suppress BAX-triggered PCD has proven a valuable initial screen for pathogen effectors capable of suppressing defense-associated PCD (Abramovitch et al., 2003; Dou et al., 2008; Wang et al., 2011a).

As a first step toward elucidating the molecular basis for colonization of apple by *V. mali*, we identified and characterized the *V. mali* repertoire of candidate effectors. Out of 70 randomly selected candidate effector proteins (CEPs), seven were able to suppress BAX-induced PCD in *Nicotiana benthamiana.* Furthermore, functional characterization of VmEP1 revealed that this candidate effector is a true virulence factor of *V. mali*. Taken together, the candidate effectors identified here provide valuable information for the study of the *V. mali*–apple interaction. Suppression of effector-triggered immunity (ETI) by this necrotrophic pathogen provides new insights into the interaction of a necrotrophic fungus and its host plant.

## Materials and Methods

### Strains and Culture Conditions

*Valsa mali* strain 03–8 is a stock culture of the Laboratory of Integrated Management of Plant Diseases at the College of Plant Protection, Northwest A&F University, Yangling, PRC (Available on request). Cultures were maintained on potato dextrose agar (PDA) medium with a layer of cellophane at 25°C in the dark. *Agrobacterium tumefaciens* strain GV3101 used for molecular cloning and agro-infiltration experiments was cultured on Luria-Bertani medium at 28°C. *N. benthamiana* plants were maintained at 25°C with 16 h illumination per day.

### Construction of *V. mali* cDNA Libraries

Total RNA was extracted using the RNeasy Micro kit (Qiagen, Shenzhen, PRC) according to the manufacturer’s protocol from (a) *V. mali* mycelium grown on PDA medium for 3 days, and (b) apple twigs of *Malus domestica* borkh. cv. ‘Fuji’ inoculated with *V. mali* mycelium [3 days post inoculation (dpi)]. First strand cDNA was synthesized by the RevertAid^TM^ First Strand cDNA Synthesis Kit (Fermentas, Shenzhen, PRC) according to the manufacturer’s protocol.

### Sequence Analyses

The secretome of *V. mali* was obtained from the sequenced genome in our previous study (Yin et al., 2015). The Whole-Genome Shotgun project for *V. mali* has been deposited at DDBJ/EMBL/GenBank under the accession JUIY01000000. CEPs were defined as extracellular proteins with no known function (*e*-value > 1e–5). Cysteine content was calculated using the pepstats program from the EMBOSS package (http://emboss.bioinformatics.nl/). Markov clustering analysis was performed using tribe-MCL (Enright et al., 2002). The known effector motifs RXLR (Kale et al., 2010), [L/I]xAR (Godfrey et al., 2010), YxSL[R/K] (Saunders et al., 2012), [R/K]VY[L/I]R (Ridout et al., 2006), and [Y/F/W]xC (Dodds et al., 2009) were searched for using the fuzzpro program from the EMBOSS package. Nuclear localization signals (NLSs) were predicted with NLStradamus (Ba et al., 2009). *De novo* motif search was performed using MEME (Bailey et al., 2009).

### Plasmid Constructs

Targeted genes were amplified from the cDNA library using high-fidelity TransStart^^®^^ FastPfu DNA polymerase (TransStart, Beijing, PRC). For *A. tumefaciens* infiltration assays in *N. benthamiana*, PCR products were digested with the appropriate restriction enzymes and ligated into the PVX vector pGR106 (Giraldo and Valent, 2013). Primers used for PCR are listed in Supplementary Table S1. All plasmids were confirmed by sequencing.

### *Agrobacterium tumefaciens* Infiltration Assays

Agro-infiltration assays were carried out following the previously described procedure (Dou et al., 2008). For transient expression, *A. tumefaciens* strain GV3101 carrying an expression plasmid (pGR106:effector) was grown in LB medium containing kanamycin (50 μg/ml). Cells were resuspended in 10 mM MgCl_2_ (pH 5.6) and cell density was adjusted to an OD_600_ = 0.4. Bacteria were infiltrated through a little nick with a syringe to the upper leaves of 4-week-old *N. benthamiana* plants. *A. tumefaciens* cells carrying the *Bax* gene (pGR106:*Bax*) were infiltrated into the same site just subsequently, or 16 h later. As control, plants were infiltrated with *A. tumefaciens* carrying an empty PVX vector. Cell death symptoms were evaluated and photographed 3–4 days past infiltration. Each assay was performed in triplicate.

### RNA Extraction and Transcript Level Analysis

To measure transcript level of candidate effector genes by qRT-PCR, apple tissue infected with *V. mali* strain 03–8 was sampled at 0, 6, 12, 24, 36, and 48 hours post inoculation (hpi). Total RNA was extracted using the RNAeasy^^®^^ Plant mini kit (Qiagen, Shenzhen, PRC) following the recommended protocol. First-strand cDNA was synthesized using an RT-PCR system (Promega, Madison, WI, USA) following the manufacturer’s instructions. SYBR green qRT-PCR assays were performed to analyze transcript levels. *V. mali* housekeeping gene *G6PDH* was used as endogenous control (Yin et al., 2013). Data from three biological replicates were used to calculate the mean and standard deviation. Primers used for qRT-PCR were listed in Supplementary Table S3.

### Generation of *VmEP1* Mutants

A typical reaction assembling three components using the hygromycin B phosphotransferase gene (*hph*) as a selective marker. The *hph* gene was amplified with primers, HPH-F (5′-GGCTTGGCTGGAGCTAGTGGAGGTCAA-3′ and HPH-R 5′-AACCCGCGGTCGGCATCTACTCTATTC-3′) from pBIG2RHPH2-GFP-GUS. The upstream (∼1,100 bp) and downstream (∼1,400 bp) flanking sequences of *VmEP1* were amplified using primer pairs VmEP1-1F/2R and VmEP1-3F/4R, respectively. Then the deletion cassette was generated by double-joint PCR as described (Yu et al., 2004), using the primer pair VmEP1-CF/CR. For generating deletion mutants, the *VmEP1* gene-replacement construct was transformed into protoplasts of *V. mali* as previously described (Gao et al., 2011). Putative deletion mutants were verified by PCR using four primer pairs (VmEP1-5F/6R, VmEP1-7F/H855R, VmEP1-H856F/8R, and H850/H852) to detect target gene (∼850 bp), upstream (∼1,100 bp), and downstream (∼1,400 bp) region, and the *hph* gene (∼750 bp), respectively. Subsequently, deletion mutants were confirmed by Southern blot hybridization using the DIG DNA Labeling and Detection Kit II (Roche, Mannheim, Germany) according to the manufacturer’s instructions. Primers used for gene deletion are listed in Supplementary Table S2.

### Complementation of the Δ*VmEp1* Mutant

A fragment containing the entire *VmEp1* gene without the termination codon and its promoter (∼1.5 kb) was amplified with primers VmEp1-FL2-F/R (5′-cgactcactatagggcgaattgggtactcaaattggTTTATCTCAATCGCCTCGTT-3′ and 5′-caccaccccggtgaacagctcctcgcccttgctcacGTCTACCGAACATGTCTGTGG-3′), and cloned into plasmid pFL2 by the yeast gap repair approach (Bruno et al., 2004; Zhou et al., 2012). The resulting construct, pFL2-VmEp1, was transformed into protoplasts of the Δ*VmEp1* mutants 74# and 85#. Δ*VmEp1*/*VmEp1* transformants were confirmed by PCR using the primer pair VmEP1-5F/6R. The Δ*VmEp1*/*VmEp1* complemented transformants 74#-1 and 85#-1were selected for phenotypic analysis.

### Vegetative Growth, Conidiation, and Pathogenicity of Mutants

Vegetative growth and conidiation was examined at three and 40 days, respectively. For our pathogenicity assay, detached apple twigs of *Malus domestica* borkh. cv. ‘Fuji’ were inoculated with *VmEP1* deletion mutants, Δ*VmEp1*/*VmEp1* complementation mutants, and wild type as described (Oliva et al., 2010). All treatments were performed with at least ten replicates, and all experiments were repeated three times. Data were analyzed by Student’s *t*-test using the SAS software package (SAS Institute, Cary, NC, USA).

### Epifluorescence Microscopy

To measure the level of colonization by the *VemEP1*-deletion mutants, boundary-zones (∼0.5 cm^2^) of inoculated leaves were sampled at 60 hpi and treated with 1 M KOH solution for 15 min at 121°C. After cooling to room temperature, the samples were washed in distilled water twice and then stained in a staining solution (0.067 M K_2_HPO_4_ + 0.05% aniline blue) according to Hood and Shew (1996). Leaf samples were examined under a Zeiss epifluorescence microscope (excitation 485 nm, dichronic mirror 510 nm, barrier 520 nm). Wild type strain 03–8 was used as control. All treatments were performed with at least five replicates, and all experiments were repeated three times.

## Results

### Characterization of Candidate Effector Proteins of *V. mali*

**FIGURE 1:**
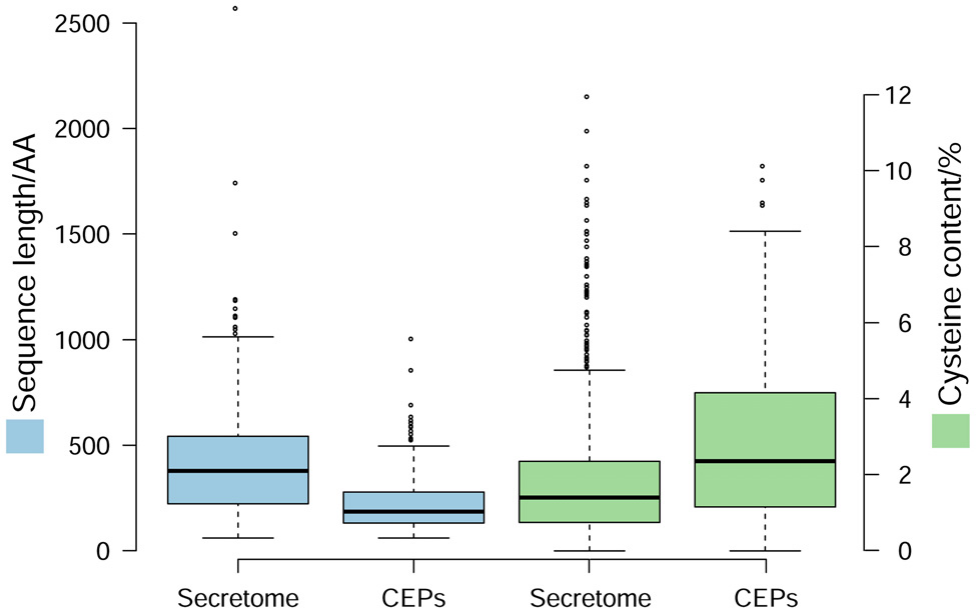
**Characteristics of candidate effector proteins (CEPs) in *Valsa mali*.** Box-plot shows sequence length and cysteine content of CEPs compared with those of the total secretome.

Considering that pathogen effector repertoires are typically lineage-specific, we defined CEPs as predicted extracellular proteins with no known function (*e*-value threshold >1e–5). Among the 779 secreted proteins predicted from the *V. mali* genome (Yin et al., 2015), we identified 193 CEPs, of which 101 are *V. mali*-specific. Analysis of amino acid sequences revealed that *V. mali* CEPs have several commonly known properties of effector proteins. They have shorter sequence length relative to non-CEPs, with an average of 233 amino acids, and are also cysteine rich (**Figure 1**). Markov clustering suggests that there is no obvious expansion of *V. mali* CEP families, and most families contain only one member.

Search for conserved motifs in these 193 CEPs showed that 71 of them contain a total of 97 Y/F/WxC motifs, 22 contain 26 L/IxAR motifs, and seven contain a single RxLR motif each. No additional conserved motifs were identified by *de novo* prediction. In addition, nine CEPs are predicted to contain NLSs.

### CEPs of *V. mali* Suppress BAX-induced PCD in *N. benthamiana*

**FIGURE 2:**
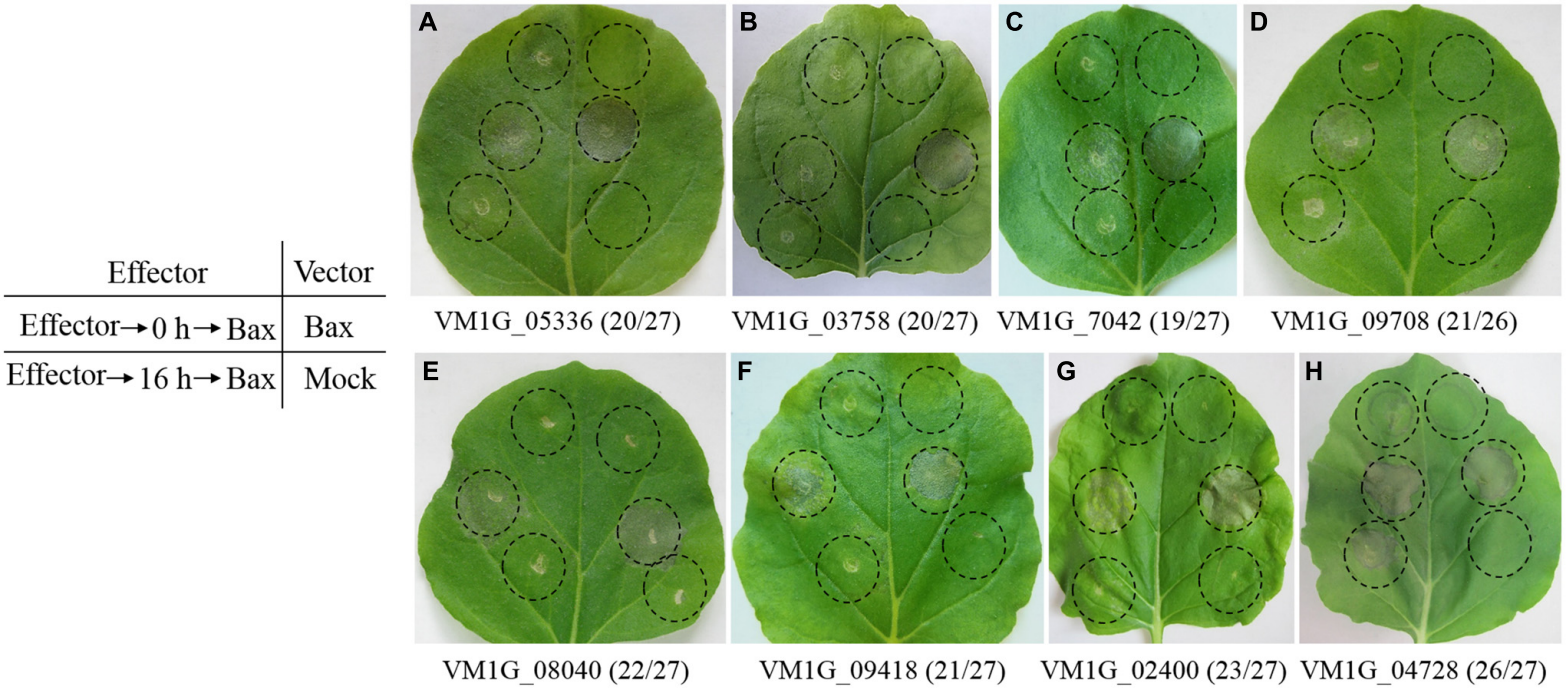
**Symptoms of transient expression of CEPs in *Nicotiana benthamiana* leaves after agro-infiltration.** **(A–G)** Seven VmEPs suppress BAX-induced cell death. **(H)** Most CEPs, like VM1G_04728, could not suppress BAX-induced PCD. Leaves were infiltrated with *A. tumefaciens* containing a PVX vector, either empty, or carrying the *Bax* gene, or the candidate effector genes within the regions indicated by the dashed lines. Photos were taken four to 5 days after the last infiltration. Numbers, for instance 20/27, indicate that 20 out of 27 times infiltrated leaves showed the same symptoms.

Phytopathogen effectors often induce a phenotype upon over-expression *in planta*, reflecting their virulence activity. To investigate the function of the CEPs of *V. mali*, we tested the ability of CEPs to induce cell death, or to suppress BAX-induced PCD in *N. benthamiana*. Based on transient over-expression in *N. benthamiana* performed for 70 randomly selected CEPs, seven *V. mali* Effector Proteins (VmEPs) significantly suppressed BAX-induced PCD. Others had little or no effect on suppressing PCD (**Figure 2**). In addition, all 70 CEPs tested could not induce cell death in *N. benthamiana*. Of these 7 VmEPs, VM1G_05336 is *V. mali*-specific and the others are hypothetical proteins that have homologs in GenBank NR database. Intriguingly, VmEP1 (VM1G_02400) contained a HeLo domain (**Table 1**) that is exclusively in the fungal kingdom and is similar as other cell death and apoptosis-inducing domains (Fedorova et al., 2005).

**Table 1:** Sequence information of *Valsa mali* effector proteins (VmEPs).

Gene_ID	Accession No.	Description	Length/AA	Cysteine content	Pfam domain
VM1G_02400	KR868746	Hypothetical protein	370	8	HeLo (PF14479)
VM1G_03758	KR868747	Hypothetical protein	134	2	NA
VM1G_05336	KR868748	NA	101	2	NA
VM1G_07042	KR868749	Hypothetical protein	330	4	NA
VM1G_08040	KR868750	Hypothetical protein	272	1	Peroxidase 2 (PF01328)
VM1G_09418	KR868751	Hypothetical protein	205	2	NA
VM1G_09708	KR868752	Hypothetical protein	285	3	NA

### Transcription Level of *V. mali* Candidate Effector Genes

**FIGURE 3:**
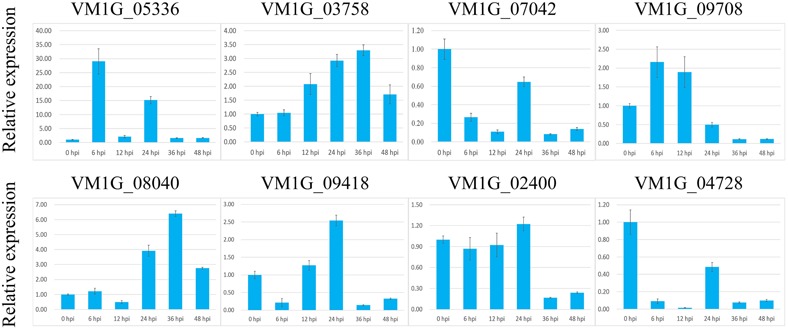
**Relative expression level of *VmEps* at 0, 6, 12, 24, 36, and 48 hours post inoculation (hpi) using reference gene *G6PDH* for normalization.** Results are presented as a mean fold change in relative expression compared to the 0 hpi sampling stage. All experiments were repeated three times. Error bars indicate SEM.

Fungal effector proteins are generally characterized by specific expression during invasion of plant cells. The expression levels of the 7 VmEP genes identified above were assayed by qRT-PCR with housekeeping gene *G6PDH* as control (**Figure 3**). Result showed that 5 of the 7 VmEP genes were up-regulated (fold change >2) during infection. Particularly, the *V. mali*-specific VM1G_05336 was remarkably induced at 6 and 24 hpi.

### Candidate Effector VmEP1 is a Virulence Factor of *V. mali*

**FIGURE 4:**
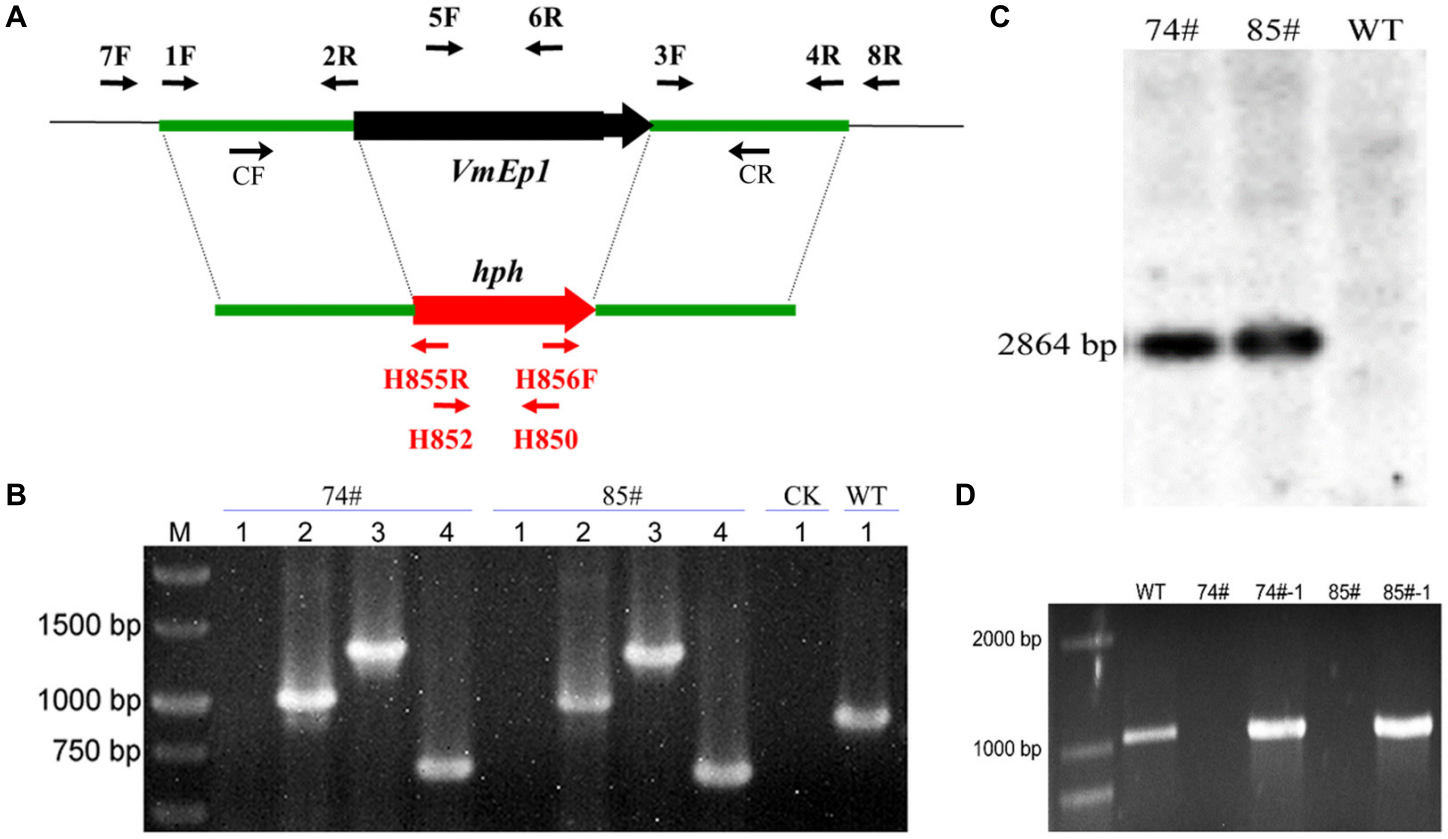
**Generation and identification of *VmEp1* deletion and complementation mutants. (A)** Gene replacement strategy of *VmEp1*. Primer pairs 1F/2R and 3F/4R used for WT amplicon. The hygromycin-resistance cassette (*hph*) is denoted by the large red arrow. Primer binding sites are indicated by black arrows (see Supplementary Table S2 for primer sequences). **(B)** Identification of *VmEp1* knockout mutants by PCR analysis. 1–4 primer pairs: (1) VmEP1-5F/6R (targeted gene), (2) VmEP1-7F/H855R upstream region), (3) VmEP1-H856F/8R (downstream region), and (4) H850/H852 (*hph* cassette). **(C)** Southern blot hybridization analysis of *VmEp1* mutants using primer H850/H852. **(D)** Identification of *VmEp1* complementation mutants by PCR analysis using primer pair VmEP1-5F/6R.

**FIGURE 5:**
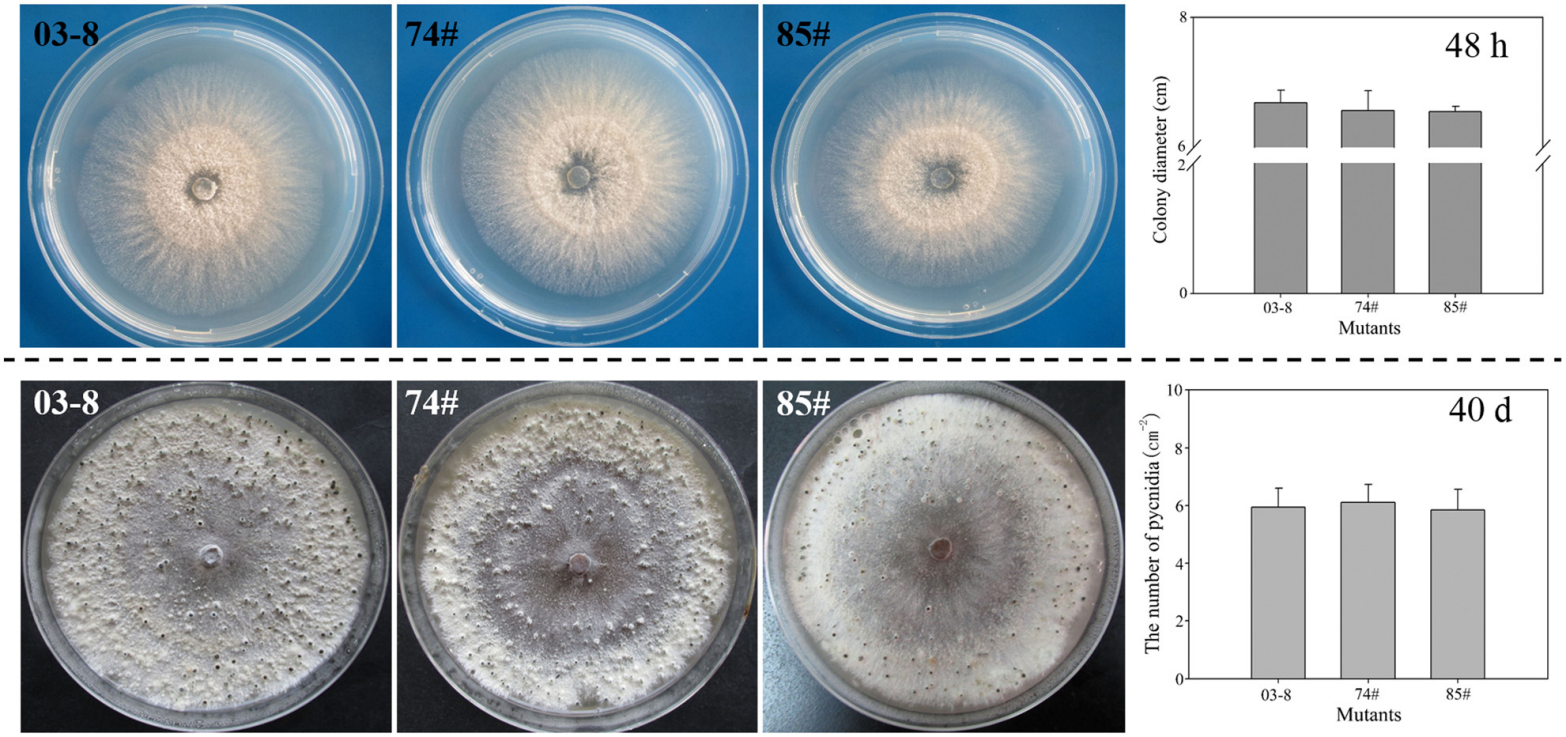
**Impact of *VmEp1* deletion on vegetative growth and conidiation.** Wild-type (03–8), and *VmEp1* deletion mutants (74#) and (85#) were grown on PDA for 48 h or 40 days at 25°C. Colony diameter and number of mature pycnidia were counted. Bars indicate standard deviation of the mean of 30 individual dishes. The experiment was repeated three times.

**FIGURE 6:**
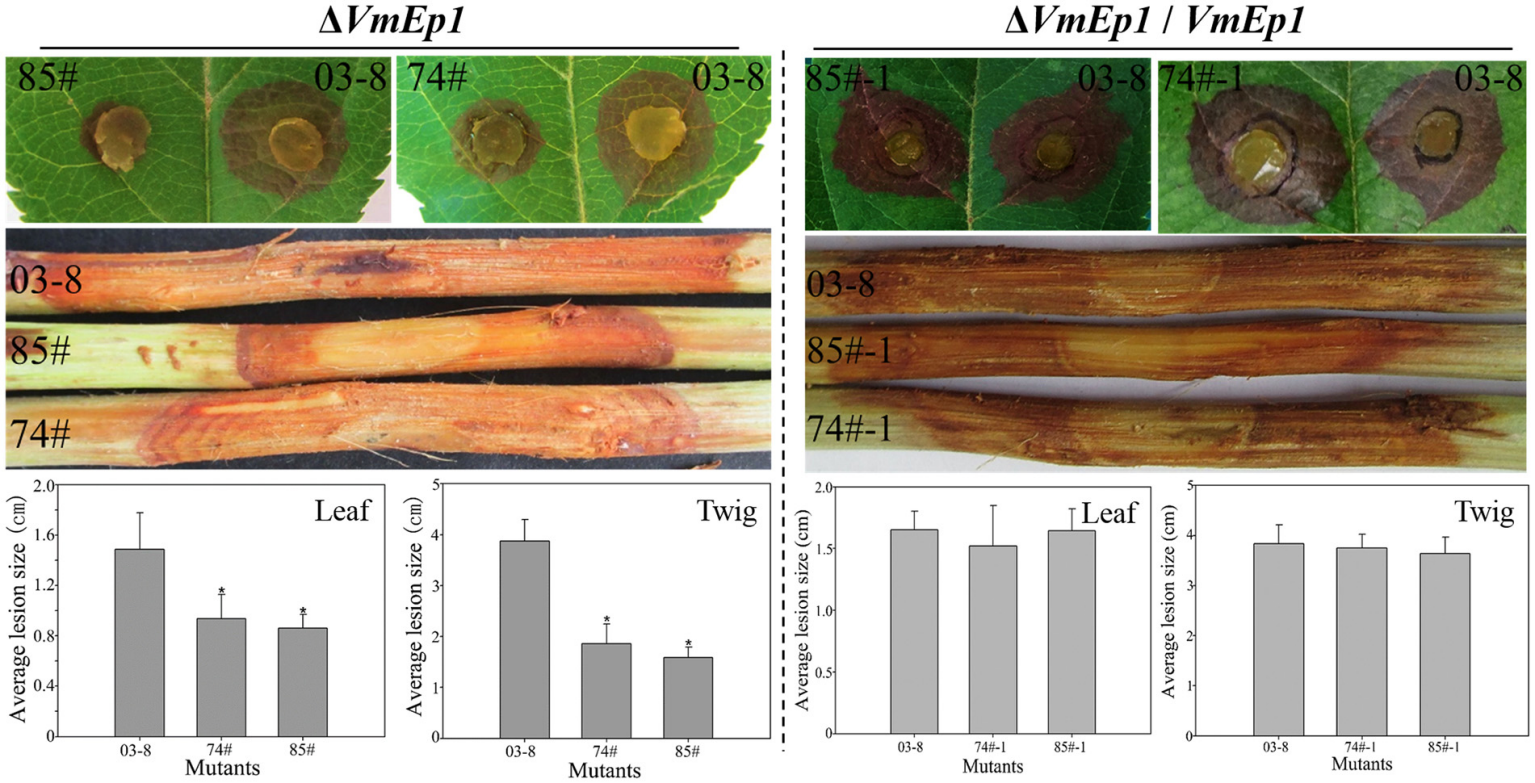
**Pathogenicity of mutants.** Wild-type (03–8), *VmEp1* deletion mutants (74#) and (85#), and complementation mutants (74#-1) and (85#-1) were inoculated on leaves and twigs of *Malus domestica* borkh. cv. ‘Fuji’ for 60 h, or 5 days, respectively. Asterisk represents a significant difference in pathogenicity (*P* < 0.05). Bars indicate standard deviation of the mean of 30 individual host plants. The experiment was repeated three times.

**FIGURE 7:**
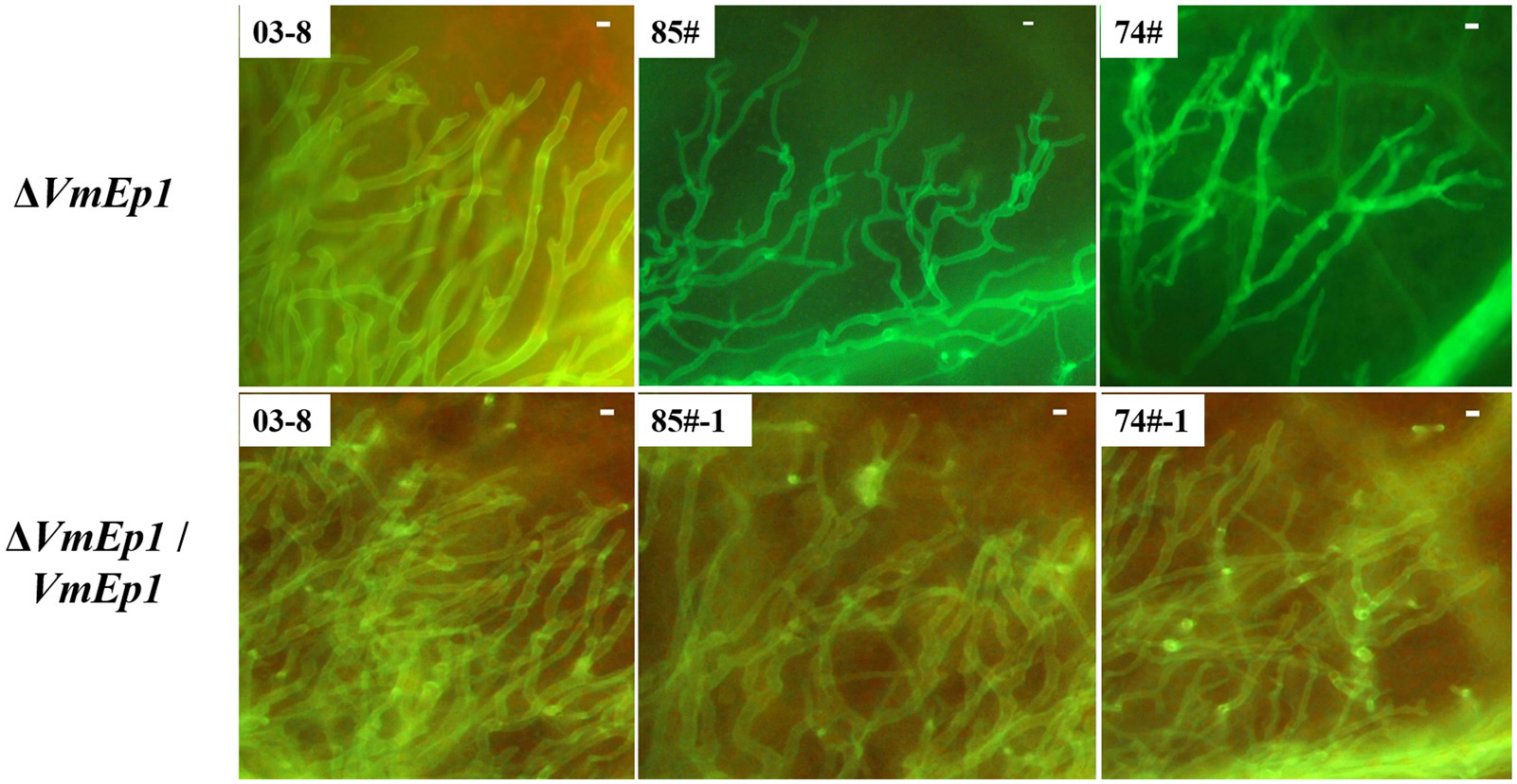
**Epifluorescence microscopy of tissue colonization during infection.** Boundary-zones (∼0.5 cm^2^) of inoculated leaves were sampled at 60 hpi. All treatments were performed with at least five replicates, and all experiments were repeated three times. Bar = 10 μm.

To investigate the function of VmEPs, the putative necrosis-inducing protein VmEP1 (VM1G_02400) and the *V. mali*-specific VM1G_05336 were chosen for further study. Gene deletion was performed using a gene replacement strategy (**Figure 4A**). However, only deletion mutants of *VmEP1* were obtained. Among the resulting 125 hygromycin-resistant transformants of *VmEP1*, two were identified by PCR analysis (**Figure 4B**). These two *VmEp1* deletion mutants (74# and 85#) were further confirmed by Southern hybridization (**Figure 4C**). Complementation of both mutants using a *VmEp1* expressing plasmid was again confirmed by PCR using the primer pair VmEP1 5F/6R (**Figure 4D**). *VmEp1* deletion mutants showed no effect on vegetative growth and conidiation on PDA medium (**Figure 5**). Intriguingly, pathogenicity assays showed that both deletion mutants had significantly reduced virulence on apple twigs and leaves (**Figure 6**). Δ*VmEp1*/*VmEp1* complemented mutants 74#-1 and 85#-1 exhibit similar virulence as the wild type isolate 03–8 (**Figure 6**). Results from epifluorescence microscopy show that deletion mutants have reduced mycelia growth within the leaf compared to the wild type which can be rescued by introducing the complementing plasmids (**Figure 7**). These results indicate that *VmEp1* is undoubtedly involved in virulence of *V. mali*.

## Discussion

In the genome of *V. mali*, we previously identified 193 genes encoding secreted proteins with no known function (Yin et al., 2015). In this study, we identified seven *V. mali* Effector Protein (VmEP) genes by a functional screen of these proteins, based on a virus *in planta* over-expression system. We showed that transient expression of each of these seven VmEPs significantly suppressed BAX-induced PCD in *N. benthamiana*, five genes of which were up-regulated during infection. While the ability to suppress BAX-triggered PCD is a valuable initial screen for pathogen effectors (Wang et al., 2011a), for it physiologically resembles defense-related HR (Lacomme and Santa Cruz, 1999), this result strongly suggests that *V. mali* expresses secreted proteins during plant infection, which can suppress effector-triggered plant immunity (ETI) responses in the host.

Effectors of necrotrophic pathogens interact with their host in a gene-for-gene relationship to initiate disease, which leads to ETS (Jones and Dangl, 2006; Oliver and Solomon, 2010; Wang et al., 2014). The host specific toxins secreted by *Cochliobolus victoriae* are translocated into plant cells to interact with specific corresponding host proteins to promote host cell death (Donofrio and Wilson, 2014; Kuhn and Panstruga, 2014). Likewise, the proteinaceous host specific toxin *Ptr*ToxA produced by *P. tritici-repentis* targets a host chloroplastic protein, which disrupts the photosynthetic capacity and triggers PCD (Stergiopoulos et al., 2013; Petre and Kamoun, 2014). Indeed, most of the identified effectors of necrotrophic pathogens are found to promote host cell death (Wang et al., 2014). However, because we screened these effector candidates on non-host *N. benthamiana*, seven out of 70 examined candidate effectors of *V. mali* suppress BAX-induced PCD and not a single one was found to induce PCD in *N. benthamiana*. Considering that many effectors of necrotrophic pathogen are host-specific toxins, it is necessary to examine ability of these CEPs to induce necrosis on hosts to determine whether they could be proteinaceous toxins.

Recently, the classic necrotrophic fungus *S. sclerotiorum* was found to have a biotrophic phase at the very early stage of infection (Kabbage et al., 2013). Oxalic acid secreted by this fungus can suppress host autophagy which is a defense response in its interaction with its host. Indeed, *S. sclerotiorum* suppresses the defense-related autophagy/PCD at an early stage of infection, and promotes disease-related apoptosis/PCD after infection established (Kabbage et al., 2013). This means that not all forms of cell deaths are equivalent. One type of cell death may be suppressed by a necrotrophic pathogen to inhibit plant defense responses, while another may be promoted to facilitate disease progress. The effectors identified in this study in *V. mali* probably participate in the suppression of defense-related PCD. Nevertheless, the interaction targets of these effector proteins in apple need to be determined to verify their exact roles in *V. mali*–apple interaction. Especially for VmEP1, because targeted deletion of *VmEP1* gene results in a significant reduction in virulence.

As a necrotrophic fungus, *V. mali* is thought to contain virulence factors that induce cell death. Particularly, the impressive arsenal of plant cell wall degrading enzymes and secondary metabolites may account for the severe necrosis observed on apple bark (Yin et al., 2015). Deletion of six pectinase genes significantly reduced virulence of *V. mali* (Yin et al., 2015). Phytotoxic small polypeptides secreted by the closely related peach canker pathogens *Leucostoma persoonii* and *L. cincta* can only induce stem necrosis on host plants, reflecting a host specific characteristic (Svircev et al., 1991). In addition, *V. mali* also possesses homologs of necrosis-inducing factors including NPP1, Ecp2, and Epl (Yin et al., 2015). Likewise, these potential virulence factors also need to be functionally verified.

## Author Contributions

ZL and ZY contributed equally to this work as first authors. LH designed and managed the project. ZL, YF, and MX performed the experimental work. ZY performed all the computational analysis. ZL, ZY, ZK, and LH wrote the paper.

## Conflict of Interest Statement

The review editor Weixing Shan declares that, despite being affiliated with the same institution as authors Zhiyuan Yin, Xu Ming, and ZhenPeng Li, the review process was carried out objectively. The authors declare that the research was conducted in the absence of any commercial or financial relationships that could be construed as a potential conflict of interest.
